# The profile of the Diploma in Anaesthesia in South Africa

**DOI:** 10.4102/jcmsa.v4i1.333

**Published:** 2026-05-13

**Authors:** Nabeelah Moola, Brian Gardner, Moses M. Kebalepile

**Affiliations:** 1 Department of Anaesthesiology, School of Clinical Medicine, University of the Witwatersrand, Johannesburg, South Africa; 2 Department of Anaesthesiology, Faculty of Medicine and Health Sciences, Walter Sisulu University, Mthatha, South Africa; 3 Education and Assessment unit, Colleges of Medicine of South Africa, Cape Town, South Africa

**Keywords:** post-graduate training, diploma in anaesthesia, career pathways, professional development, clinical competence

## Abstract

**Background:**

South Africa faces a shortage of anaesthetists, particularly in rural areas. The Diploma in Anaesthesia (DA) was created to address this gap, but its role may now serve as a preparatory step for specialist training. This study assessed DA graduates’ career paths, perceived value of the diploma, geographical distribution and self-perceived clinical confidence.

**Methods:**

This cross-sectional study was conducted via virtual response to a questionnaire using Research Electronic Data Capture (REDCap^®^). Data were analysed using descriptive statistics.

**Results:**

One hundred and ninety-two diplomates responded. Most DA candidates practised anaesthesia, with 44% working as DA anaesthetists and 41% pursuing the specialist route. Most candidates (60%) practised in urban areas, particularly in Gauteng (49%) and the Western Cape (17%). Eighty-six per cent of respondents viewed the DA as a step within the Fellowship of the College of Anaesthetists (FCA) curriculum. Most respondents felt the DA tested their competence (86.5%), safety (88%) and skills and knowledge (94.8%).

**Conclusion:**

Most diplomates practise within the urban setting, potentially limiting the diploma’s value in enriching the quality of anaesthesia provided in rural areas. High perceptions of the DA’s value in testing competence and safety suggest its continued relevance in anaesthesia training. The view that the DA is a preparatory step within the FCA process indicates a need for ongoing evaluation of the DA curriculum and its alignment with current workforce demands.

**Contribution:**

This research will contribute to the optimisation of the DA, enhancing its utility for its intended purpose or improving its structure to better align with its current application.

## Introduction

Safe anaesthesia is a critical component of delivering high-quality surgical care. Healthcare systems in developed and developing countries face a significant shortage of anaesthetists, posing a challenge to ensuring optimal patient outcomes during surgical procedures. The World Federation of Societies of Anaesthesiologists recommends a ratio of five anaesthetic providers per 100 000 people.^1^ South Africa has an estimated ratio of 2.48 anaesthetists per 100 000 people. Of these, most specialist anaesthesiologists practice in urban areas. Rural communities may, therefore, experience a greater scarcity of skilled personnel necessary for appropriate and safe perioperative care.^2,3^

In 1974, the Colleges of Medicine of South Africa introduced the Diploma in Anaesthetics (DA) to increase the number of trained anaesthesia providers.^4^ Historically, the DA aimed to train the general practitioner (GP) to provide high-quality, safe anaesthesia.^5^ Diplomates are expected to have a basic understanding of relevant pharmacology, physiology, physics, equipment and clinical measurements. They must accurately assess patients and provide anaesthesia in various clinical settings across a broad patient population.^5^ A 1999 evaluation of career pathways of anaesthesia diplomates found that over 70% of them remained active in anaesthesia, of whom a third pursued speciality training. Among the GP anaesthetists, one third practised in small towns or rural locations. The conclusion at the time was that the DA exam fulfilled its purpose.^6^

The medical fraternity has subsequently grown, with greater numbers of both specialist and GP doctors.^7^ Perioperative care has become more complex encompassing a broader range of procedures, patient profiles and management approaches.^8^ Registrar programmes are more competitive, and GPs may use the DA to enter a specialist programme. If most diplomates are currently using the DA as a foundation towards career paths other than general practitioner anaesthesia practice, then a review of the DA curriculum and scope of practice would need to be considered. This would have implications for how the College of Anaesthesia structures the eligibility criteria and format of the DA exam.

This study aimed to determine how the DA contributes to creating an anaesthesia workforce; test whether the DA continues to develop safe GP anaesthetists; and locate where GP anaesthetists practice in South Africa.

## Research methods and design

This study was a prospective cross-sectional descriptive study. It was conducted virtually using Research Electronic Data Capture (REDCap^®^) Version 13.4.12, hosted by the University of the Witwatersrand.^9^

All successful candidates of the Colleges of Medicine of South Africa (CMSA) DA from January 2016 to December 2023 were invited to participate. The CMSA identified potential participants from its database and contacted them via email. Candidates who received diplomas from other institutions and those unwilling to provide informed consent were excluded.

The sample size was determined from the number of successful candidates over the specified period (*n* = 1220, from January 2016 to December 2023). A minimum sample size, calculated using EpiInfo Version 7, was established to be 266 people. This number was calculated to generalise to the study population, using a confidence interval of 95% and an error margin of five percent.

The survey was based on a previous study conducted in 1999 at the University of Cape Town by Gordon and James.^6^ Permission was obtained from the authors to use and modify the survey as needed. The questionnaire was loaded onto REDCap^®^. A link to the electronic survey was distributed electronically to diplomates via email from the CMSA with an information and informed consent statement.

Continuous data were tested for normality, and appropriate descriptive statistics were used to report measures of central tendency. Proportions were used to describe categorical variables.

### Ethical considerations

This study was accepted by the CMSA. Ethical clearance to conduct this study was obtained from University of the Witwatersrand and Human Research Ethics Committee (No. M240509).

## Results

Altogether, 192 out of 266 people completed the survey, giving a response rate of 72.2%. The median age of participants was 33 (interquartile range [IQR] 4).

### Participants’ geographical location

The geographical distribution of respondents showed that most (95.1%) were practising within South Africa, of which Gauteng was the most well-represented province (49.4%) (Table 1).

**TABLE 1:** Geographical distribution of respondents across provinces.

Location	*n*	%
Eastern Cape	12	7.14
Gauteng	83	49.4
Mpumalanga	4	2.3
Free State	3	1.7
Northern Cape	5	2.9
North West	1	0.6
KwaZulu-Natal	16	9.5
Limpopo	1	0.6
Western Cape	30	17.8
Outside SA	13	7.7

Note: Question unanswered (*n* = 24; 12.5%); Number of respondents (*n* = 168; 87.5%).

SA, South Africa.

Of the 83 DA anaesthetists who practise in South Africa, 50 (60%) are based in cities, 24 (29%) in towns and 9 (11%) in rural areas.

### The career paths of successful Diploma in Anaesthesia candidates

Most diplomates (92.6%) continued working in anaesthesia after completing their DA, either as DA anaesthetists (44.4%), anaesthetic registrars (38.1%) or specialist anaesthetists (3.7%). A minority (3.7%) worked abroad and some candidates chose not to specify their current anaesthetic practise (2.7%). Additionally, 86% of respondents reported that more than 80% of their daily practice was primarily anaesthesia.

### Value of the Diploma in Anaesthesia and skill development

A large proportion of respondents (86%) viewed the DA as a step within the FCA curriculum, 7% did not share this view, and 6% were unsure.

**FIGURE 1:**
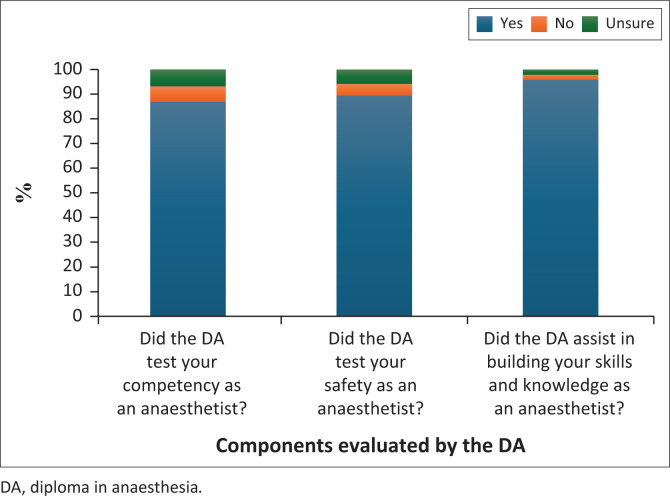
Candidates’ perceptions of how the diploma in anaesthesia evaluates their competency, safety, skills and knowledge.

Most respondents (166; 86.5%, *p* = 0.081) felt the DA tested their competence; 169 (88%, *p* = 0.025) felt it tested safety and 182 (94.8%, *p* = 0.432) skills and knowledge. This was only significant for safety (Figure 1).

**FIGURE 2:**
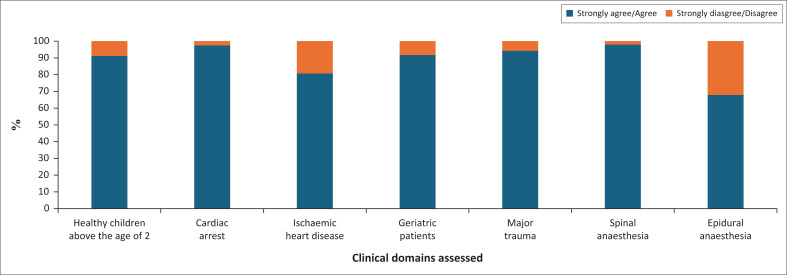
Candidates’ confidence in skills in managing clinical scenarios.

Most respondents agreed that the DA equipped them with adequate skills and knowledge to deal with the following: Healthy children over the age of 2 years, cardiac arrest, ischaemic heart disease, geriatric patients, major trauma, spinal and epidural anaesthesia (Figure 2). There was no significant difference in how prepared candidates felt in these scenarios (Chi-square test, *p* = 0.432).

**FIGURE 3:**
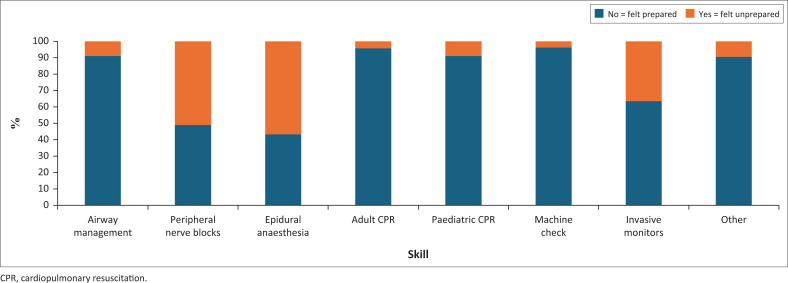
Perceptions of how well the diploma in anaesthesia prepared candidates for specific skills required in daily practice.

Respondents most frequently felt that they were under trained in peripheral nerve blocks (*n* = 98; 51.6%) and epidural anaesthesia (*n* = 109; 56.8), compared to airway management (*n* = 175; 91.1%), adult cardiopulmonary resuscitation (*n* = 184; 95.8%), paediatric cardiopulmonary resuscitation (*n* = 175; 91.1%) and machine checks (*n* = 185; 96.4%) (Figure 3).

**FIGURE 4:**
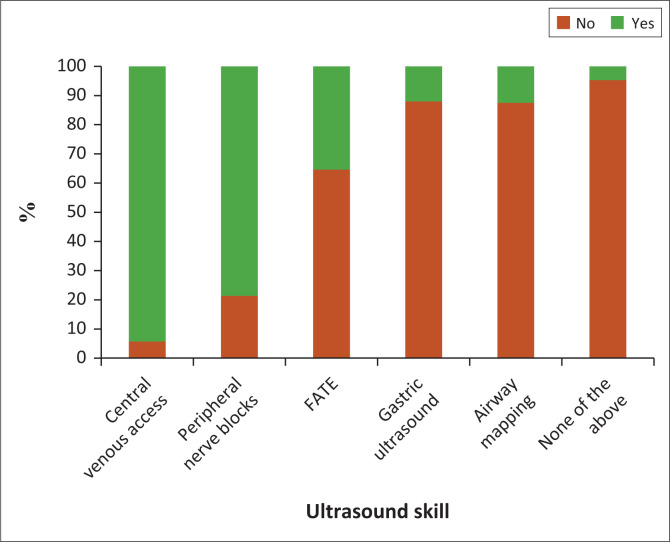
Candidates’ comfort with applications of ultrasound technology.

Participants felt the DA improved their ultrasound skills (*p* < 0.001). They were most frequently comfortable using ultrasound for central venous access and peripheral nerve blocks. They were most frequently uncomfortable using ultrasound for focused assessed transthoracic echocardiography (FATE), gastric ultrasound and airway mapping (Figure 4).

## Discussion

Most DA candidates chose to work in anaesthesia after completing their DA, with 44% practising as anaesthetists and 41% pursuing the speciality route, either currently in an FCA programme or having already obtained an FCA. This demonstrates an increased movement towards specialisation compared to the past, where around a third (32.4%) of diplomates chose to obtain an FCA.^6^ The decline in DA graduate retention may also reflect a global structural shift within medicine, in which generalist and intermediate professional roles are increasingly displaced by specialist and subspecialist career trajectories.^10^ A similar trend may be seen in South Africa, where most (93%) of medical students at a single university have expressed an intention to specialise, although this may not be generalisable nationally.^11^ While the current data did not directly test this assumption, a similar argument has been described before, in which the authors argue that professional advancement, credentialling systems and institutional incentives increasingly favour specialised professional identity, a dynamic that may also help to explain why intermediate qualifications are less likely to serve as long-term career endpoints.^10^ This shift risks undermining the DA’s original purpose of supplying South Africa with safe GP anaesthetists who are widely distributed to meet national healthcare needs.

The proportion of DA graduates pursuing a speciality may be higher than the reported 41%. The DA is becoming a prerequisite for entry into speciality anaesthesia programmes in South Africa, as reflected in registrar post advertisements.^12^ This trend is further supported by findings from the current study, where 86% of respondents perceived the DA as a stepping stone to the FCA curriculum. This represents a three-and-a-half-fold increase from the approximately 25% of diplomates who held this view in 1999.^6^ Given that this study surveyed recent DA graduates, an even greater proportion may now be pursuing further anaesthesia education. Undoubtedly, the DA helps entry-level registrars become more proficient. However, if candidates sitting the exam increasingly view the DA as a preparatory qualification, this trend may hinder capacity building in rural settings.

The distribution of DA anaesthetists in our study population in South Africa demonstrates a marked concentration in urban areas, with 60% (*n* = 50) practising in cities, 29% (*n* = 24) in towns and 11% (*n* = 9) in rural regions. Previous studies have shown similar findings.^6^ Gauteng accounted for the largest proportion of DA anaesthetists, followed by the Western Cape, likely reflecting practitioner preference or surgical capacity, as urban areas have more surgical beds and operating theatres.^13^ This urban–rural disparity is particularly significant in South Africa where geographic isolation, insufficient infrastructure and workforce shortages constrains healthcare access.^14^ Without sufficient anaesthesia coverage, efforts to expand surgical services in underserved areas are limited, and rural communities may experience delays or reduced access to essential care.^15^ A more detailed examination of the barriers influencing the distribution of DA anaesthetists, and possibly anaesthesiologists, could help inform targeted interventions to improve the equitable distribution of anaesthesia care across South Africa.

The high perception of the DA’s value in testing competence, safety and skills suggests its continued relevance. Diplomates demonstrated confidence in managing most given clinical scenarios. While statistically insignificant (*p* = 0.432), they reported lower confidence levels in handling epidural anaesthesia and ischaemic heart disease than in other clinical situations. This finding aligns with previous research and may reflect a reduced use of epidural services in the patient population typically managed by diplomates rather than indicating a notable area for improvement.^6^

A small proportion (8.9%) of candidates felt that the DA did not prepare them adequately for paediatric cardiopulmonary resuscitation. Although this result is not statistically significant, the finding that a proportion of anaesthesia providers report discomfort with paediatric cardiopulmonary resuscitation in the theatre environment is nonetheless concerning and merits further attention. Paediatric cardiopulmonary resuscitation is currently assessed in the DA examination, indicating that the curriculum may benefit from enhanced focus or targeted practical training to further strengthen candidate’s competence and confidence.

The DA improved ultrasound skills in the central venous access and peripheral nerve block domains, although confidence was lower in FATE, gastric ultrasound and airway mapping. Given the rapid advances in ultrasound technology and its growing role in anaesthesia, training in ultrasound technology could be enhanced, potentially as mandatory competencies within the DA curriculum.^16^

Several limitations should be considered when interpreting these findings. Selection bias may be present, as non-responders may differ systematically from responders, potentially underrepresenting rural practitioners. The small rural subsample (*n* = 9) constrains the generalisability of rural-specific findings. Self-reported measures of confidence and safety may not accurately reflect actual clinical competence. The absence of a comparator group or objective data on patient outcomes further limits evaluation of clinical impact. The sample size is skewed towards younger practitioners (median age = 33), limiting insight into long-term career trajectories. Although the study achieved a relatively high response rate (72.2%), the final sample size (*n* = 192) was lower than the minimum estimated sample size of 266. This shortfall may have reduced the statistical power of the study, particularly for subgroup and secondary analyses, thereby limiting the ability to detect smaller or moderate associations. Consequently, some non-significant findings, including those presented in Figure 1, should be interpreted with caution, as they may reflect limited power rather than the true absence of an effect. Nevertheless, the sample remains informative for the descriptive and exploratory objectives of the study.

## Conclusion

The DA was established to bridge the gap between general practitioners and specialist anaesthetists, aiming to ensure the delivery of safe, high-quality anaesthetic care to a vulnerable and expanding patient population in South Africa. Since the inception of the DA, the anaesthesia workforce landscape has evolved with registrar programmes becoming more competitive and the DA now being seen as a preparatory pathway to the FCA curriculum. Many diplomates pursue specialisation, and most practise in urban areas. While the DA has value in skills and knowledge development, it may play a lesser role in enhancing the quality and safety of anaesthesia provided in towns and rural areas. Future research should investigate factors influencing career progression towards specialisation and the barriers to rural practice.
